# Übersetzung der 2018 EULAR Empfehlungen zu körperlicher Aktivität von Menschen mit entzündlich-rheumatischen und degenerativen Erkrankungen ins Deutsche und sprachliche Validierung im deutschsprachigen Raum mit medizinischen Fachpersonen

**DOI:** 10.1007/s00393-021-01078-0

**Published:** 2021-10-11

**Authors:** Uta Kiltz, David Kiefer, Jürgen Braun, Anne-Kathrin Rausch-Osthoff, Susanne Herbold, Meike Klinger, Agnes Kocher, Valerie Nell-Duxneuner, Stefan Reichenbach, Tanja Stamm, Patricia Steffens-Korbanka, Karin Niedermann

**Affiliations:** 1 Rheumazentrum Ruhrgebiet, Ruhr-Universität Bochum, Claudiusstr. 45, 44649 Herne, Deutschland; 2 Institut für Physiotherapie, Zürcher Hochschule für Angewandte Wissenschaften, Winterthur, Schweiz; 3 Departement Gesundheitswissenschaften, Fachhochschule Campus Wien, Wien, Österreich; 4 Institut für Pflegewissenschaft, Medizinische Fakultät, Departement Public Health, Universität Basel, Basel, Schweiz; 5 Medizinischer Dienst, Österreichische Gesundheitskasse, Wien, Österreich; 6 Ludwig Boltzmann Institute for Arthritis and Rehabilitation, Wien, Österreich; 7 Klinik für Rheumatologie und Immunologie, Inselspital, Universitätsspital Bern, Bern, Schweiz; 8 Section for Outcomes Research, Center for Medical Statistics, Informatics and Intelligent Systems, Medical University of Vienna, Wien, Österreich; 9 Rheumapraxis an der Hase, Osnabrück, Deutschland

**Keywords:** EULAR Empfehlungen, Körperliche Aktivität, Arthritis, Deutsche Übersetzung, Sprachliche Validierung, EULAR recommendations, Exercise, Arthritis, German translation, Linguistic validation

## Abstract

**Hintergrund:**

Regelmäßige Bewegung und spezifisches Training sind wichtige Bausteine in der Therapie rheumatischer Erkrankungen, weil ein gesundheitlicher Nutzen für die Patient*innen nachgewiesen ist. Basierend auf den internationalen Empfehlungen der WHO für Gesunde, geben die „2018 EULAR Empfehlungen zu körperlicher Aktivität von Menschen mit entzündlich-rheumatischen und degenerativen Erkrankungen“ erstmals evidenzbasierte Empfehlungen zu Gestaltung, Durchführung und Implementierung von Bewegungsübungen bei diesen Patient*innen.

**Ziel:**

Übersetzung ins Deutsche und sprachliche Validierung in Deutschland, Österreich und der Schweiz.

**Methoden:**

Eine professionelle Übersetzung der EULAR Bewegungsempfehlungen ins Deutsche wurde durch deutschsprachige Experten*innen aus allen 3 Ländern überarbeitet. Die Validierung erfolgte in einem Feldtest mit Rheumatolog*innen, Ergo- und Physiotherapeut*innen, Pflegefachpersonen und medizinischen Fachangestellten aus der Rheumatologie. In den 3 Ländern wurden jeweils 8 strukturierte Interviews zu Verständlichkeit, Wortwahl, Vollständigkeit und Umsetzbarkeit durchgeführt. Die Experten*innen diskutierten die Änderungsvorschläge, bis jeweils ein Konsens erreicht wurde. Zuletzt gaben sie den Grad ihrer Zustimmung zu der finalen Übersetzungsversion an.

**Ergebnisse:**

Die professionelle Übersetzung wurde substanziell überarbeitet. Aufgrund der Ergebnisse der Feldtests wurden verschiedene Änderungen einzelner Worte sowie Umformulierungen zur besseren Verständlichkeit vorgenommen. Der Grad der Zustimmung lag mit durchschnittlichen Bewertungen zwischen 10 (SD 0,0) und 8,9 (SD 1,5) insgesamt sehr hoch.

**Diskussion:**

Die vorliegende sprachlich validierte deutschsprachige Version der 2018 EULAR Bewegungsempfehlungen kann und soll dazu beitragen, Fachpersonal darin zu unterstützen, körperliche Aktivität bei Menschen mit entzündlich-rheumatischen und degenerativen Erkrankungen zu fördern.

## Abstract (en)

**Background:**

Regular physical activity is beneficial for people with rheumatic diseases and one of the cornerstones in its management. Based on the international recommendations of the World Health Organization for the general population, the “2018 EULAR recommendations for physical activity in people with inflammatory arthritis and osteoarthritis” provide evidence-based recommendations on the prescription, performance, and implementation of physical activity exercises in this population for the first time.

**Aim:**

Translation of the 2018 EULAR recommendations into German and linguistic validation in Germany, Austria and Switzerland.

**Methods:**

A professional translation of the EULAR recommendations into the German language was performed and revised by German-speaking experts from all three countries. The translation was validated by healthcare professionals consisting of rheumatologists, occupational therapists, physiotherapists, nurses, and medical assistants in a field test. In each of the three countries, eight structured interviews were conducted on comprehensibility, wording, completeness, and feasibility. The experts then discussed changes until consensus was reached and indicated the level of agreement with the final translation.

**Results:**

The translation of the EULAR recommendations was substantially revised. Based on the results of the cognitive test, formulations were adapted in order to increase comprehensibility. The level of agreement between 10 (SD 0.0) and 8.9 (SD 1.5) was very high.

**Discussion:**

The final German version of the EULAR recommendations is comprehensible and accepted across all three German-speaking countries. It can help to improve the structure and clarity of the handling of physical activity and promote physical activity for healthcare providers and patients.

## Hintergrund

Die Durchführung von regelmäßiger körperlicher Aktivität und spezifischem Training ist ein wichtiger Baustein in der Therapie entzündlich-rheumatischer und degenerativer Erkrankungen. Patient*innen, die regelmäßig körperlich aktiv sind, klagen weniger häufig über Schmerzen, haben eine besser erhaltene körperliche Funktionsfähigkeit, fühlen sich weniger häufig müde und weisen eine bessere Kontrolle ihrer Krankheitsaktivität auf [1, 4, 18]. Neben der Verbesserung klinischer Symptome bei Patient*innen, die sich regelmäßig körperlich bewegen, wurde in mehreren Studien eine Verbindung zwischen entzündlichen Prozessen im Körper und hormonähnlichen Botenstoffen, den Myokinen, festgestellt. Diese gehören zur Gruppe der Interleukine, sie werden von der Muskulatur bei Bewegung und Kontraktion ausgeschüttet und haben zum Teil antientzündliche Eigenschaften [2].

Trotz vorliegender Evidenz sind Patient*innen mit rheumatischen und degenerativen Erkrankungen im Vergleich zu Gesunden im Allgemeinen aber weniger körperlich aktiv [6, 9, 19]. Die „2018 EULAR Empfehlungen zu körperlicher Aktivität von Menschen mit entzündlich-rheumatischen und degenerativen Erkrankungen“ (im Folgenden „EULAR Bewegungsempfehlungen“ genannt) sind mit dem Ziel publiziert worden, evidenzbasierte Empfehlungen zu Gestaltung, Durchführung und Implementierung von körperlicher Aktivität für diese Patient*innen zu formulieren [17]. Basierend auf dieser Publikation, wurde 2019 eine laienverständliche Version auf Englisch veröffentlicht [14]. Die EULAR Bewegungsempfehlungen basieren auf einer systematischen Literaturrecherche zur Evidenz von körperlicher Aktivität für Patient*innen mit rheumatoider Arthritis (RA), Spondyloarthritiden (SpA inklusive der axialen SpA und der Psoriasisarthritis) sowie Hüft- und Kniegelenkarthose (HKA) [16].

Die internationale und interprofessionelle Expert*innengruppe setzte sich aus 22 europäischen Experten*innen (6 Mediziner inklusive 3 Rheumatolog*innen, einem orthopädischen Chirurgen, 9 Physiotherapeut*innen (PT), einem Psychologen, einem Ergotherapeuten, einer Pflegefachperson und einer Bewegungswissenschaftlerin) zusammen. Insgesamt wurden 4 übergeordnete Prinzipien und 10 Empfehlungen formuliert, die sich an medizinisches Fachpersonal, individuelle Patient*innen, Patient*innenorganisationen und politische Entscheidungsträger*innen richten. Für die Versorgung der Patient*innen stellen die EULAR Bewegungsempfehlungen insgesamt einen Meilenstein zur Anerkennung des Nutzens und der Förderung von körperlicher Aktivität und spezifischem Training bei Patient*innen mit entzündlich-rheumatischen und degenerativen Erkrankungen dar.

Körperliche Aktivität beinhaltet jede durch die Skelettmuskulatur hervorgebrachte Bewegung, die den Energieverbrauch über den Ruhezustand ansteigen lässt. Körperliche Aktivität umfasst spezifisches Training, Sport sowie körperliche Aktivität als Teil von Alltag, Beruf, Freizeit und aktiver Fortbewegung [3, 8]. Spezifisches Training ist eine Subkategorie von körperlicher Aktivität. Spezifisches Training ist eine geplant, strukturiert und regelmäßige Handlung mit dem Ziel, körperliche Fitness zu verbessern oder zu erhalten [3, 8]. In diesem Manuskript und in den Empfehlungen umfasst der Begriff „körperliche Aktivität“ auch „spezifisches Training“. Wo dies besonders betont werden soll, wird der Begriff spezifisches Training explizit verwendet. Zudem sind auch Techniken zur Unterstützung der Veränderung des Bewegungsverhaltens und der langfristigen Adhärenz wichtig [10, 13]. Um die gesundheitlichen Vorteile von körperlicher Aktivität in der Allgemeinbevölkerung zu fördern, hat die Weltgesundheitsorganisation (WHO) und das American College of Sports Medicine (ACSM) international anerkannte Bewegungsempfehlungen für Gesunde veröffentlicht (im Folgenden „Bewegungsempfehlungen für Gesunde“) [8, 15]. Zudem definierte das ACSM Prinzipien für das Training in verschiedenen Fitnessdimensionen, wie z. B. hinsichtlich Häufigkeit, Intensität und Zeitaufwand (s. Tab. 2).

Alle internationalen und nationalen Empfehlungen für RA, SpA sowie Arthrose empfehlen die regelmäßige Durchführung von spezifischem Training [7, 11, 12, 20]. Die EULAR Bewegungsempfehlungen gehen über eine reine Propagierung von körperlicher Aktivität und strukturiertem Training hinaus und geben auch spezifische Hinweise, wie körperliche Aktivität in der Standardversorgung angeboten und durch das Behandlungsteam unterstützt werden soll. Dies beinhaltet auch, welche individuellen Ziele vereinbart werden, welche Barrieren und Förderfaktoren angesprochen werden sollen und letztlich welche Möglichkeiten es für die Durchführung von körperlicher Aktivität gibt. Diese EULAR Bewegungsempfehlungen sollen nicht nur Gesundheitsdienstleister*innen, sondern alle Menschen dabei unterstützen, körperliche Aktivität langfristig zu unterstützen und zu fördern.

Die Übersetzung erfolgte mit dem Ziel, die Verbreitung und klinische Umsetzung der EULAR Bewegungsempfehlungen mit einer gemeinsamen Version in den deutschsprachigen Ländern Deutschland, Österreich und der Schweiz zu fördern und damit möglichst vielen Gesundheitsfachpersonen den Zugang zu ermöglichen [21]. In dieser Publikation wird erstmals die international konsentierte Übersetzung der EULAR Bewegungsempfehlungen für entzündlich-rheumatische und degenerative Erkrankungen ins Deutsche einschließlich der Feldtestung für die sprachliche Validierung vorgestellt.

## Methode

Es handelt sich um ein zweiphasiges Projekt im deutschsprachigen Raum (Deutschland, Österreich, Schweiz): In der ersten Phase bearbeitete die Autor*innengruppe eine professionelle Übersetzung der EULAR Bewegungsempfehlungen ins Deutsche. In einer zweiten Phase wurde die bearbeitete Übersetzung in einem Feldtest mit medizinischem Fachpersonal, welches an der Übersetzung nicht beteiligt war, sprachlich validiert. In einem parallelen Projekt wurde die lai*innenverständliche Version der EULAR Bewegungsempfehlungen in Deutsch übersetzt und validiert und wird ebenfalls in dieser Fachzeitschrift publiziert werden.

### Übersetzung der EULAR Bewegungsempfehlungen ins Deutsche

Die professionelle Übersetzung der EULAR Bewegungsempfehlungen sowie der Bewegungsempfehlungen für Gesunde wurde im Februar 2020 durch die Autor*innengruppe aus dem deutschsprachigen Raum, bestehend aus Rheumatolog*innen, Ergo- und Physiotherapeut*innen (ET/PT), Pflegefachpersonen sowie medizinischen Fachangestellten (MFA) aus der Rheumatologie überarbeitet.

### Sprachliche Validierung der deutschen Übersetzung der EULAR Bewegungsempfehlungen

Die Validität der bearbeiteten Übersetzung wurde anschließend anhand von kognitiven Debriefing-Interviews durchgeführt. Dazu wurden strukturierte Interviews mit medizinischen Fachpersonen aus den Berufsgruppen Rheumatologie, ET, PT, Pflege und MFA durchgeführt. Dabei wurde ein strukturierter Fragebogen zu Verständlichkeit, Wortwahl, Vollständigkeit, Verwendbarkeit und Umsetzung der Übersetzung ausgefüllt. Die Teilnehmenden konnten dazu ihre Zustimmung mittels dichotomer Antwortoption ja/nein angeben. Bei Nicht-Zustimmung konnten zu jedem Aspekt eigene Vorschläge und Ergänzungen eingebracht werden. Soziodemografische Angaben zu Alter, Geschlecht, Berufserfahrung in der Rheumatologie (Jahre) der Teilnehmenden wurden erfasst.

Die Ergebnisse der Interviews wurden durch die Experten*innengruppe ausgewertet und alle Kommentare der Teilnehmenden zusammen analysiert. Die Formulierungen/Wortwahl der Empfehlungen wurden ggf. angepasst, ohne aber die Aussage der Empfehlungen zu verändern. Der Grad der Zustimmung wurde von der Experten*innengruppe nach der Validierung in anonymen Verfahren per E‑Mail auf einer numerischen Rating-Skala (NRS) von 0–10 (10 höchste Zustimmung) für jede einzelne Empfehlung bewertet.

## Ergebnisse

### Übersetzung

Die professionelle Übersetzung wurde Satz für Satz mit der englischen Version verglichen, sprachliche Auffälligkeiten und v. a. länderspezifische Verwendung von Begrifflichkeiten wurde durch die Experten*innengruppe, mit Mitgliedern aus der Schweiz, Österreich sowie Deutschland, diskutiert und ein Konsens zur Übersetzung der 10 EULAR Bewegungsempfehlungen, der 4 übergeordneten Prinzipien (Tab. 1) und der ACSM-Definitionen (Tab. 2) gefunden.

**Table 1:** 

**Übergeordnete Prinzipien**		**Evidenzgrad**	**Grad der Zustimmung, Mittelwert (SD)**		
1.	Körperliche Aktivität ist Teil eines generellen Konzepts zur Optimierung der gesundheitsbezogenen Lebensqualität	–	10,0 (0)		
2.	Körperliche Aktivität bringt gesundheitliche Vorteile für Menschen mit rheumatoider Arthritis (RA)/Spondyloarthritis (SpA)/Hüft‑/Kniegelenkarthrose	–	9,9 (0,3)		
3.	Die allgemeinen Empfehlungen zu körperlicher Aktivität für Gesunde umfassen die 4 Bereiche kardiorespiratorische Fitness, Muskelkraft, Beweglichkeit und neuromotorische Leistungsfähigkeit. Sie gelten genauso für Menschen mit RA/SpA/Hüft- und Kniegelenkarthrose, weil sie sicher angewendet werden können	Anwendbarkeit 4 Sicherheit 1A, 1B	9,4 (0,9)		
4.	Die Planung von körperlicher Aktivität erfordert eine gemeinsame Entscheidungsfindung zwischen Gesundheitsdienstleistern und Menschen mit RA/SpA/Hüft- und Kniegelenkarthrose unter Berücksichtigung der Präferenzen, Fähigkeiten und Ressourcen der jeweiligen Person	–	9,8 (0,4)		
**Empfehlung**		**Evidenzkategorie**	**Empfehlungsstärke**	**Grad der Zustimmung, Mittelwert (SD)**	**Grad der Zustimmung in der Originalpublikation, Mittelwert (SD)**
1.	Die Förderung von körperlicher Aktivität in Übereinstimmung mit den allgemeinen Empfehlungen zu körperlicher Aktivität sollte ein integraler Bestandteil der Standardversorgung während des gesamten Erkrankungsverlaufs von Menschen mit RA/SpA/Hüft- und Kniegelenkarthrose sein	1B	A	10,0 (0)	9,81 (0,39)
2.	Alle an der Versorgung von Menschen mit RA/SpA/Hüft- und Kniegelenkarthrose beteiligten Gesundheitsdienstleister sollten Verantwortung für die Förderung von körperlicher Aktivität übernehmen. Sie sollten zusammenarbeiten und notwendige Überweisungen vornehmen, um sicherzustellen, dass angemessene Interventionen zur Förderung von körperlicher Aktivität erfolgen	4	D	9,5 (0,8)	9,14 (0,98)
3.	Interventionen zur Förderung der körperlichen Aktivität sollten von Gesundheitsdienstleistern vorgenommen werden, die kompetent sind, diese bei Menschen mit RA/SpA/Hüft- und Kniegelenkarthrose durchzuführen	4	D	9,1 (1,1)	8,86 (1,48)
4.	Gesundheitsdienstleister sollten bei Menschen mit RA/SpA/Hüft- und Kniegelenkarthrose die Art, Intensität, Häufigkeit und Dauer der aktuell ausgeübten körperlichen Aktivität mit standardisierten Methoden evaluieren, um festzustellen, welche der 4 Bereiche der allgemeinen Empfehlungen zur körperlichen Aktivität gezielt zu verbessern sind	3	C	8,9 (1,1)	9,05 (1,04)
5.	Allgemeine und krankheitsspezifische Kontraindikationen für körperliche Aktivität sollten identifiziert und bei der Förderung der körperlichen Aktivität berücksichtigt werden	4	D	9,6 (0,8)	9,1 (1,41)
6.	Interventionen zur Förderung der körperlichen Aktivität sollten klare personalisierte Ziele haben, die fortlaufend, vorzugsweise durch eine Kombination aus subjektiven und objektiven Messinstrumenten evaluiert werden (inklusive Selbstbeobachtung, wenn angemessen)	4	D	9,0 (1,8)	9,05 (1,25)
7.	Allgemeine und krankheitsspezifische Barrieren und Förderfaktoren in Bezug auf die Durchführung von körperlicher Aktivität sollten identifiziert und adressiert werden. Barrieren und Förderfaktoren beziehen sich auf Kenntnisse, soziale Unterstützung, Symptomkontrolle und Selbstregulierung	3–4	C–D	9,6 (1,0)	9,19 (1,13)
8.	Wenn individuelle Anpassungen an die allgemeinen Empfehlungen zur körperlichen Aktivität erforderlich sind, sollten diese auf einer umfassenden Erhebung von körperlichen, sozialen und psychologischen Faktoren inklusive Fatigue, Schmerz, Depression und Krankheitsaktivität basieren	4	D	8,9 (1,5)	9,25 (0,86)
9.	Gesundheitsdienstleister sollten Interventionen zur Förderung von körperlicher Aktivität planen und durchführen. Das schließt Techniken zur Verhaltensänderung inklusive Selbstbeobachtung, Zielsetzung, Aktionsplanung, Feedback und Problemlösung ein	1A	A	9,6 (0,8)	9,48 (0,79)
10.	Gesundheitsdienstleister sollten in Übereinstimmung mit den Präferenzen der Person mit RA/SpA/Hüft- und Kniegelenkarthrose verschiedene Arten der Durchführung von körperlicher Aktivität in Erwägung ziehen (z. B. angeleitet/nicht angeleitet, Einzel/Gruppe, Präsenz/Online, Verstärkungsstrategien etc.)	4	D	9,8 (0,6)	9,0 (1,3)

*SD* Standardabweichung

**Table 2:** 

**Empfehlungen zu körperlicher Aktivität vom American College of Sports Medicine (ACSM) und von der American Heart Association (AHA)**	
Alle gesunden Erwachsenen im Alter von 18 bis 65 Jahren sollten mindestens an 5 Tagen pro Woche 30 min lang aerobe Aktivitäten mit moderater Intensität oder an mindestens 3 Tagen pro Woche 20 min lang aerobe Aktivitäten mit hoher Belastungsintensität durchführen	
Kombinationen aus aerober Aktivität mit moderater und hoher Intensität sind ebenfalls geeignet, um diese Empfehlung zu erfüllen	
Die aerobe Aktivität soll in Einheiten von ≥10 min^a^ Dauer durchgeführt werden, um die insgesamt 30 min zu erreichen	
Jede erwachsene Person sollte mindestens 2 Tage pro Woche körperliche Aktivitäten ausüben, die die Muskelkraft und Ausdauer erhalten oder steigern	
Wegen der Dosis-Wirkung-Beziehung von körperlicher Aktivität und Gesundheit (je mehr desto besser) können Personen über die empfohlene Mindestmenge an körperliche Aktivität hinausgehen, wenn sie ihren allgemeinen Fitnesszustand weiter verbessern, ihr Risiko für chronische Erkrankungen und Folgeschäden verringern und/oder eine ungesunde Gewichtszunahme verhindern möchten	
**Kardiorespiratorisches (aerobes) Training**	
*Häufigkeit*	Mindestens 5 Tage pro Woche moderates Training oder ≥3 Tage pro Woche intensives Training oder eine Kombination aus moderatem und intensivem Training an mindestens 3 bis 5 Tagen pro Woche wird empfohlen
*Intensität*	Mäßige und/oder hohe Intensität wird für die meisten Erwachsenen empfohlen. Leichte bis moderate Intensität kann bei untrainierten Personen sinnvoll sein
*Dauer*	30–60 min pro Tag (150 min pro Woche)^b^ gezieltes moderates Training oder 20–60 min pro Tag (75 min)^b^ intensives Training oder eine Kombination davon wird für die meisten Erwachsenen empfohlen. Ein Training von mindestens 20 min pro Tag (mindestens 150 min pro Woche) kann insbesondere bei zuvor eher inaktiven Menschen sinnvoll sein
*Art und Weise*	Regelmäßige und gezielte Bewegungen, bei denen große Muskelgruppen beteiligt sind und die kontinuierlich und rhythmisch sind
*Umfang*	Mindestens 500–1000 MET^c^ min pro Woche werden empfohlen. Eine Erhöhung der Schrittzahl um mindestens 2000 Schritte pro Tag auf eine tägliche Anzahl von mindestens 7000 Schritte wird empfohlen. Auch ein Training unterhalb dieser Schwelle ist sinnvoll, wenn diese Empfehlungen nicht umgesetzt werden können oder möchten
*Schema*	Das Training kann in einer (fortlaufenden) Einheit durchgeführt oder auf mehrere Einheiten pro Tag (von ≥10 min) aufgeteilt werden, je nach gewünschter Dauer und Umfang des Trainings. Trainingsblöcke von ≥10 min können bei sehr dekonditionierten Personen Vorteile bringen. Intervalltraining ist ebenfalls möglich
*Steigerung*	Eine schrittweise Steigerung des Trainingsvolumens durch Anpassung der Trainingsdauer, -frequenz und/oder -intensität ist sinnvoll, bis das gewünschte Trainingsziel (Aufrechterhaltung) erreicht ist. Die schrittweise Steigerung und individuelle Anpassung kann die Adhärenz verbessern sowie das Auftreten von Verletzungen des Bewegungsapparates und von unerwünschten kardiovaskulären Ereignissen verringern
**Krafttraining**	
*Häufigkeit*	Jede größere Muskelgruppe sollte an 2 bis 3 Tagen pro Woche trainiert werden
*Intensität*	60–70 % des 1 RM^d^ bei moderater bis hoher Intensität für Anfänger bis fortgeschrittene Anfänger zur Verbesserung der Muskelkraft
	≥80 % des 1 RM bei hoher bis sehr hoher Intensität für trainingserfahrene Personen
	40–50 % des 1 RM bei sehr geringer bis geringer Intensität für ältere Personen, die mit dem Training beginnen, zur Verbesserung der Muskelkraft
	40–50 % des 1 RM bei geringer bis sehr geringer Intensität kann für die Verbesserung der Kraft bei eher inaktiven Menschen zu Beginn des Krafttrainings sinnvoll sein
	≤50 % des 1 RM bei leichter bis moderater Intensität zur Verbesserung der Muskelkraftausdauer
	20–50 % des 1 RM bei älteren Erwachsenen zur Verbesserung der Muskelkraft
*Zeitaufwand*	Für die Wirksamkeit wurde keine spezifische Trainingsdauer ermittelt
*Art und Weise*	Es werden Kraftübungen empfohlen, an denen jede größere Muskelgruppe beteiligt ist. Für solche Übungen kann mit verschiedenen Trainingsgeräten und/oder Körpergewichten gearbeitet werden
*Wiederholungen*	8 bis 12 Wiederholungen, um die Muskelkraft und Muskelleistung bei den meisten Erwachsenen zu verbessern
	10 bis 15 Wiederholungen, um die Muskelkraft bei Personen mittleren und höheren Alters, die mit dem Training beginnen, zu verbessern
	15 bis 20 Wiederholungen, um die Muskelkraftausdauer zu verbessern
*Einheiten*	2 bis 4 Übungssätze werden empfohlen, um die Muskelkraft und Muskelleistung bei den meisten Erwachsenen zu verbessern. Ein einziger Satz von Kraftübungen kann besonders bei älteren und unerfahrenen Trainierenden wirksam sein. Mindestens 2 Sätze verbessern die Muskelkraftausdauer
*Schema*	Pausenintervalle von 2–3 min zwischen jedem Übungssatz
	Eine Pause von ≥48 h zwischen den Trainingseinheiten für eine einzelne Muskelgruppe wird empfohlen
*Steigerung*	Eine allmähliche Steigerung über höheren Widerstand und/oder mehr Wiederholungen pro Satz und/oder Häufigkeit werden empfohlen
**Beweglichkeitstraining**	
*Häufigkeit*	≥2 bis 3 Tage pro Woche verbessern das Bewegungsausmaß der Gelenke, wobei die größten Fortschritte durch tägliches Training erzielt werden
*Intensität*	Dehnen bis zum Gefühl von Spannung oder leichtem Schmerz
*Zeitaufwand*	Für die meisten Erwachsenen wird eine statische Dehnung von 10–30 s empfohlen Bei älteren Menschen kann eine Dehnung von 30–60 s einen größeren Nutzen bringen
	Bei einem PNF^e^-Stretching sollte die Kontraktionsdauer etwa 3–6 s betragen bei maximal 20–75 % der Maximalkraft, gefolgt von einer 10–30 s dauernden assistiven Dehnung
*Art und Weise*	Eine Serie von Beweglichkeitsübungen für die wichtigsten Muskel-Sehnen-Verbindungen wird empfohlen
	Statisches Dehnen (aktiv oder passiv), dynamisches Dehnen, ballistisches Dehnen und PNF^e^-Dehnen sind wirksam
*Umfang*	Ein realistisches Ziel sind insgesamt 60 s Dehnung für jede Beweglichkeitsübung
*Schema*	Es wird empfohlen, jede Beweglichkeitsübung 2‑ bis 4‑mal zu wiederholen. Die Beweglichkeitsübung ist am effektivsten, wenn der Muskel durch leichte bis moderate Aktivität oder passive Methoden wie feuchte Wärmepackung oder heiße Bäder erwärmt wird
*Steigerung*	Methoden zur optimalen Steigerung sind nicht bekannt
**Neuromotorisches Training (z.** **B. Gleichgewicht, Agilität, Koordination, Gang)**	
*Häufigkeit*	≥2 bis 3 Tage pro Woche werden empfohlen
*Intensität*	Eine effektive Intensität für das neuromotorische Training ist ungeklärt
*Zeitaufwand*	≥20–30 min pro Tag können erforderlich sein
*Art und Weise*	Älteren Menschen werden ein Training der motorischen Fertigkeiten (z. B. Gleichgewicht, Agilität, Koordination und Gang), propriozeptives Training sowie variantenreiche Bewegungsformen (z. B. Yoga) empfohlen, um die körperliche Funktionsfähigkeit zu erhalten und zu verbessern und das Sturzrisiko zu reduzieren
	Die Wirksamkeit eines neuromotorischen Trainings ist für Menschen jüngeren und mittleren Alters nicht nachgewiesen, ist allerdings wahrscheinlich nützlich
*Umfang*	Der optimale Übungsumfang, z. B. Anzahl der Wiederholungen oder Intensität, ist nicht bekannt
*Schema*	Das optimale Bewegungsmuster für das neuromotorisches Training ist nicht bekannt
*Steigerung*	Methoden zur optimalen Steigerung sind nicht bekannt

^a^Aktualisierte WHO-Empfehlungen 2020: keine Evidenz, dass <10 min nicht auch effektiv sein sollte, daher zählen auch Einheiten <10 min

^b^Aktualisierte WHO-Empfehlungen 2020: 30–60 min pro Tag (150–300 min pro Woche) gezieltes moderates Training oder 20–60 min pro Tag (75–150 min pro Woche) intensives Training oder eine Kombination davon wird für die meisten Erwachsenen empfohlen

^c^*MET* metabolisches Äquivalent

^d^*1* *RM* „one repitition maximum“, wobei 1 RM dem maximalen Gewicht oder der maximalen Kraftanstrengung entspricht

^e^*PNF* propriozeptive neuromuskuläre Fazilitation

### Validierung

Es wurden 24 Interviews mit je 6 Rheumatolog*innen, ETs, PTs, Pflegefachpersonen respektive MFAs durchgeführt und analysiert. Die demografischen Daten der Teilnehmenden sind in Tab. 3 dargestellt. Die einzelnen Kommentare wurden gruppiert und werden im folgenden Freitext zu den jeweiligen Empfehlungen kursiv aufgeführt und ggf. kommentiert. Änderungen durch die Experten*innengruppe sind im Freitext durch ein „#“ gekennzeichnet.

**Table 3:** 

	Deutschland	Österreich	Schweiz	Total
*Teilnehmer (n)*	8	8	8	24
*Geschlecht, männlich, n (%)*	5 (62,5)	3 (37,5)	2 (25)	8 (36)
*Alter, in Jahren, Mittelwert (SD)*	37,9 (9,2)	36,8 (8,2)	36,6 (10,6)	37,8 (9,1)
*Beruf*				
Rheumtolog*innen	2	2	2	6
Ergotherapeut*innen	2	2	2	6
Physiotherapeut*innen	2	2	2	6
Medizinische Fachangestellte und Pflegefachpersonal	2	2	2	6
*Berufserfahrung in der Rheumatologie Mittelwert in Jahren (SD)*	11,3 (9,6)	8,3 (8,3)	9,4 (6,1)	9,6 (7,9)

*SD* Standardabweichung

### Finale Version der Übersetzung ins Deutsche

Im Folgenden wird die finale Version der übergeordneten Prinzipien (Abschnitt *Zusammenfassende Beurteilung zu Verständlichkeit, Wortwahl, Vollständigkeit, Umsetzung und Verwendbarkeit*) und den 10 Empfehlungen zu körperlicher Aktivität (Abschnitt *Übergeordnete Prinzipien*) sowie die ACSM-Definitionen der verschiedenen Fitnessdimensionen tabellarisch (Abschnitt *EULAR Bewegungsempfehlungen*; Tab. 1 und 2) dargestellt. Der Grad der Zustimmung (Mittelwert ± Standardabweichung) der Experten*innen sowie die Angaben zur Evidenzkategorie und Stärke der Empfehlung (A–D) aus der Originalpublikation der EULAR Bewegungsempfehlungen sind in Tab. 1 zusammengefasst. Im Fließtext wird zusätzlich auf ausgewählte Aspekte der Übersetzung eingegangen.

#### Zusammenfassende Beurteilung zu Verständlichkeit, Wortwahl, Vollständigkeit, Umsetzung und Verwendbarkeit

Es wurden insgesamt 175 Kommentare durch die 24 Teilnehmenden abgegeben. Im Schnitt stimmten ≥95 % der Teilnehmenden der Übersetzung hinsichtlich Verständlichkeit, Wortwahl, Vollständigkeit, Umsetzung und Verwendbarkeit zu. Die relevanten Kritikpunkte zur Verständlichkeit der Übersetzung werden jeweils bei den einzelnen Empfehlungen wiedergegeben.

In Bezug auf Verständlichkeit und Wortwahl der Übersetzung lag eine hohe Zustimmung der Teilnehmenden vor. Die hier nicht ausführlich kommentierten Änderungen zur Verständlichkeit bezogen sich auf die Umstellung einzelner Worte sowie von Sätzen und Abkürzungen. Diese Kritikpunkte wurden in der Expert*innengruppe diskutiert und großenteils umgesetzt.

Die Teilnehmenden bewerteten die inhaltlichen Domänen als vollständig. Es wurde angemerkt, dass auch Hinweise zu einzelnen Verordnungsmöglichkeiten gegeben werden sollten (z. B. Rehasport oder Funktionstraining). Dieser Vorschlag wurde nicht umgesetzt, da sich die Verordnungsfähigkeit in den verschiedenen Ländern unterscheidet. Des Weiteren wurde kommentiert, dass neben den körperlichen, sozialen und psychologischen Faktoren inklusive Fatigue, Schmerz, Depression und Krankheitsaktivität auch auf die Domäne „Angst“ hingewiesen werden sollte. Dies wurde nicht umgesetzt, da sich der Punkt zu weit vom Text der englischen EULAR Bewegungsempfehlungen entfernt hätte. Die Frage nach der Umsetzung und Verwendbarkeit der Empfehlungen wurde ebenfalls sehr hoch bewertet. Einige kritische Kommentare bezogen sich auf länderspezifische Probleme wie z. B. der Interaktion verschiedener Berufsgruppen. Die Experten*innengruppe beschloss, länderspezifische Aspekte zur Umsetzung der EULAR Bewegungsempfehlungen grundsätzlich nicht in die deutsche Version aufzunehmen.

#### Übergeordnete Prinzipien

1. Körperliche Aktivität ist Teil eines generellen Konzepts zur Optimierung der gesundheitsbezogenen Lebensqualität.
2. Körperliche Aktivität bringt gesundheitliche Vorteile für Menschen mit RA/SpA/HKA.
3. Die Bewegungsempfehlungen für Gesunde umfassen die 4 Bereiche kardiorespiratorische Fitness, Muskelkraft, Beweglichkeit und neuromotorische Leistungsfähigkeit. Sie gelten genauso für Menschen mit RA/SpA/HKA, weil sie sicher angewendet werden können.
4. Die Planung von körperlicher Aktivität erfordert eine gemeinsame Entscheidungsfindung zwischen Gesundheitsdienstleistern und Menschen mit RA/SpA/HKA unter Berücksichtigung der Präferenzen, Fähigkeiten und Ressourcen der jeweiligen Person.

##### Kommentar zur deutschen Übersetzung der übergeordneten Prinzipien.

*Werden degenerative Veränderungen und Arthrosen im Bereich der Wirbelsäule von den Empfehlungen ausgeschlossen?*

# Degenerative Wirbelsäulenveränderungen werden in den EULAR Bewegungsempfehlungen nicht berücksichtigt, da die Datenlage nur für HKA analysiert worden ist.

*Was bedeutet „sicher und machbar“?*

# Der Satz wurde dahingehend geändert, dass die Empfehlungen für die Patient*innen sicher anwendbar sind.

#### EULAR Bewegungsempfehlungen

##### 1. EULAR Empfehlung.

Die Förderung von körperlicher Aktivität in Übereinstimmung mit den Bewegungsempfehlungen für Gesunde sollte ein integraler Bestandteil der Standardversorgung während des gesamten Erkrankungsverlaufs von Menschen mit RA/SpA/HKA sein.

##### Kommentar zur deutschen Übersetzung.

Keine Kommentare. Diese erste Empfehlung wurde von Interviewten/Teilnehmenden als allgemein verständlich beurteilt.

##### 2. EULAR Empfehlung.

Alle an der Versorgung von Menschen mit RA/SpA/HKA beteiligten Gesundheitsdienstleister*innen sollten Verantwortung für die Förderung von körperlicher Aktivität übernehmen. Sie sollten zusammenarbeiten und notwendige Überweisungen vornehmen, um sicherzustellen, dass angemessene Interventionen zur Förderung von körperlicher Aktivität erfolgen.

##### Kommentar zur deutschen Übersetzung.

Der Begriff „Gesundheitsdienstleister*innen“ ist unklar, es solle definiert werden, welche Berufsgruppen dazugehören.

# Da viele Berufsgruppen Verantwortung für die Förderung von körperlicher Aktivität haben können, wurde, auch um keine Berufsgruppen auszuschließen, bewusst auf eine Aufzählung verzichtet. Zudem gibt es in den 3 beteiligten Ländern keinen einheitlich verwendeten Begriff.

##### 3. EULAR Empfehlung.

Interventionen zur Förderung von körperlicher Aktivität sollten von Gesundheitsdienstleistern vorgenommen werden, die kompetent sind, diese bei Menschen mit RA/SpA/HKA durchzuführen.

##### Kommentar zur deutschen Übersetzung.

*Wer wird als kompetent bezeichnet?*

# Unter kompetent werden Gesundheitsdienstleister*innen beschrieben, die Expertise auf den Gebieten der körperlichen Aktivität und rheumatischen Erkrankungen haben. Eine offizielle Zertifizierung, um die Kompetenz der Gesundheitsdienstleister*innen nachzuweisen, gibt es derzeit nicht.

##### 4. EULAR Empfehlung.

Gesundheitsdienstleister*innen sollten bei Menschen mit RA/SpA/HKA die Art, Intensität, Häufigkeit und Dauer der aktuell ausgeübten körperlichen Aktivität mit standardisierten Methoden evaluieren, um festzustellen, welche der 4 Bereiche der allgemeinen Empfehlungen zu körperlicher Aktivität gezielt zu verbessern sind.

##### Kommentar zur deutschen Übersetzung.

*Die 4 Bereiche sollten aufgezählt werden.*

# Die 4 Bereiche umfassen kardiorespiratorische Fitness, Muskelkraft, Beweglichkeit und neuromotorische Leistungsfähigkeit, diese werden im Text beschrieben und in der 4. Empfehlung nicht erneut aufgezählt. Eine Tabelle zur Darstellung der 4 Bereiche wurde angefertigt (Tab. 4).

**Table 4:** 

Bereich	Art und Weise	Beispiele
*Kardiorespiratorisches (aerobes) Training*	Regelmäßige und gezielte Bewegungen, bei denen große Muskelgruppen beteiligt sind und die kontinuierlich und rhythmisch sind	Gehen, Laufen, Schwimmen, Radfahren
*Krafttraining*	Kraftübungen, an denen größere Muskelgruppen beteiligt sind. Körperliche Aktivität und Übungen, die die Kraft, Leistung, Ausdauer und Masse der Skelettmuskulatur erhöhen	Krafttraining, Widerstandstraining oder Übungen für Muskelkraft und Ausdauer
*Beweglichkeitstraining*	Flexibilitätsübungen verbessern die Fähigkeit eines Gelenks, sich über seinen gesamten Bewegungsbereich zu bewegen	Spezifische Dehnungstechniken wie statisches Dehnen (aktiv oder passiv), dynamisches Dehnen, ballistisches Dehnen, Propriozeptive neuromuskuläre Fazilitation
*Neuromotorisches Training*	Training der motorischen Fertigkeiten, koordinatives Training sowie variantenreiche Bewegungsformen, um die körperliche Funktionsfähigkeit zu erhalten und zu verbessern und das Sturzrisiko zu reduzieren	Gleichgewicht, Agilität, Koordination, Gang, Yoga

##### 5. EULAR Empfehlung.

Allgemeine und krankheitsspezifische Kontraindikationen von körperlicher Aktivität sollten identifiziert und bei der Förderung von körperlicher Aktivität berücksichtigt werden.

##### Kommentar zur deutschen Übersetzung.

*Was sind Kontraindikationen für *körperliche Aktivität*?*

# Die Förderung der körperlichen Aktivität sollte individuell erfolgen, wenn andere Erkrankungen (z. B. Herz-Kreislauf-Erkrankungen, Lungenerkrankungen, Sehschwäche, Frakturen) oder krankheitsspezifische individuelle Pathologien vorliegen (z. B. Herzbeteiligung, Halswirbelsäuleninstabilität).

##### 6. EULAR Empfehlung.

Interventionen zur Förderung von körperlicher Aktivität sollten klare personalisierte Ziele haben, die fortlaufend, vorzugsweise durch eine Kombination aus subjektiven und objektiven Messinstrumenten evaluiert werden (inklusive Selbstbeobachtung, wenn angemessen).

##### Kommentar zur deutschen Übersetzung.

*Selbstmonitoring ist schlecht verständlich.*

# Der Begriff „Selbstmonitoring“ wurde geändert in „Selbstbeobachtung“.

##### 7. EULAR Empfehlung.

Allgemeine und krankheitsspezifische Barrieren und Förderfaktoren in Bezug auf die Durchführung von körperlicher Aktivität sollten identifiziert und adressiert werden.

Barrieren und Förderfaktoren beziehen sich auf Kenntnisse, soziale Unterstützung, Symptomkontrolle und Selbstregulierung.

##### Kommentar zur deutschen Übersetzung.

*Was bedeuten in diesem Fall die Begriffe „Barrieren“ und „Förderfaktoren“?*

# In der finalen Version wurde aus einem Satz zwei gemacht, um den zweiten Satz als Erklärung hervorzuheben.

##### 8. EULAR Empfehlung.

Wenn individuelle Anpassungen an die Bewegungsempfehlungen für Gesunde erforderlich sind, sollten diese auf einer umfassenden Erhebung von körperlichen, sozialen und psychologischen Faktoren inklusive Fatigue, Schmerz, Depression und Krankheitsaktivität basieren.

##### Kommentar zur deutschen Übersetzung.

*Fatigue sollte durch ein Synonym ersetzt werden.*

# Fatigue ist eine eigenständige Bezeichnung und beinhaltet bei Patient*innen mit entzündlich-rheumatischen Erkrankungen mehr als reine Müdigkeit oder Erschöpfung und wurde daher als Begriff belassen. Die Autor*innen stimmen dem Vorschlag insofern zu, als dass sie anerkennen, dass das Wort Fatigue kein Wort aus dem deutschen Wortschatz ist. Aufgrund der Multidimensionalität des Symptomkomplexes gibt es aber auch kein adäquates Wort in Deutsch, welches als Synonym verwendet werden könnte.

##### 9. EULAR Empfehlung.

Gesundheitsdienstleister*innen sollten Interventionen zur Förderung von körperlicher Aktivität planen und durchführen. Das schließt Techniken zur Verhaltensänderung inklusive Selbstbeobachtung, Zielsetzung, Aktionsplanung, Feedback und Problemlösung ein.

##### Kommentar zur deutschen Übersetzung.

*Der Satz ist umständlich formuliert. Was ist ein Aktionsplan?*

# Der ursprüngliche Satz wurde geändert und vereinfacht. Ein Aktionsplan (im Original „action plan“) ist die Planung, wie die Verbesserung körperlicher Aktivität durchzuführen und umzusetzen ist. Der Begriff Aktionsplan wird als Begriff im Bereich der Bewegungsförderung häufig verwendet. Der Kontext des deutschen Wortes „Aktionsplan“ unterscheidet sich aber zwischen den 3 beteiligten Ländern.

##### 10. EULAR Empfehlung.

Gesundheitsdienstleister*innen sollten in Übereinstimmung mit den Präferenzen der Menschen mit RA/SpA/HKA verschiedene Arten der Durchführung von körperlicher Aktivität in Erwägung ziehen (z. B. angeleitet/nicht angeleitet, Einzel/Gruppe, Präsenz/Online, Verstärkungsstrategien etc.).

##### Kommentar zur deutschen Übersetzung.

*Der Begriff „Boosterstrategien“ ist nicht verständlich genug.*

# Anstelle „Boosterstrategien“ wurde der Begriff „Verstärkungsstrategien“ gewählt. Verstärkungsstrategien sind zu verstehen als Strategien zur Erinnerung und Auffrischung der erlernten Inhalte. Der Begriff Boosterstrategie wird als Begriff in dem Bereich der Gesundheitsförderung häufiger verwendet.

#### Bewegungsempfehlungen für Gesunde gemäß den ACSM-Definitionen

Die Bewegungsempfehlungen für Gesunde vom ACSM umfassen 4 Empfehlungen zur Art und Dosierung von körperlicher Aktivität (Tab. 2). Im Anschluss an die Empfehlungen werden die Trainings der 4 Fitnessdimensionen in Bezug auf Häufigkeit, Intensität, Dauer, Art und Weise, Umfang, Schema und Steigerung definiert.

##### Kommentar zur deutschen Übersetzung der Tab. 2.

*Die Abkürzung „MET“ sollte erklärt werden.*

*Was bedeutet „One repitition maximum“?*

*„PNF“ sollte erklärt werden.*

*Was bedeutet aerobe Aktivität oder aerobes Training?*

*Die Zeiteinheiten der WHO-Empfehlung von 2011 sind nicht mehr aktuell.*

*Was bedeutet Adhärenz?*

*Was sind Hauptmuskelgruppen?*

Einige Begrifflichkeiten warfen Verständnisfragen auf, zudem wurden einige wenige englische Abkürzungen in der Tab. 3 belassen. Diese Begrifflichkeiten werden im Folgenden erläutert.

# Das metabolische Äquivalent (MET; „metabolic equivalent of task“) wird als dimensionslose Einheit verwendet, um den Energieverbrauch eines individuellen Menschen bei verschiedenen Aktivitäten zu messen. Für die verwendeten Intensitäten (leicht, moderat, hoch) gilt allgemein als Lai*innenregel für ein moderates Training, dass das Sprechen noch möglich ist, wohingegen bei einer hohen Intensität kein Sprechen mehr möglich ist.

# „One repitition maximum“ (1 RM) beschreibt das maximale Gewicht, das eine Person nur 1‑mal in einem definierten Bewegungsbereich bewegen kann.

# Die propriozeptive neuromuskuläre Fazilitation (PNF; „proprioceptive neuromuscular faciliation“) ist ein physiotherapeutisches Konzept zur Analyse und Behandlung von Bewegungsmustern.

# Aerobe Aktivität oder aerobes Training: beinhaltet kontinuierliche und rhythmische Bewegungen bei submaximaler Ausdauerbelastung, welche alle großen Muskelgruppen mit einschließt (wie z. B. Schwimmen, Joggen).

# Adhärenz ist das konsequente Verhalten einer Person hinsichtlich eines Therapieziels („adhere“ = an etwas halten) und zeigt anders als Compliance („comply“ = befolgen) die aktive Teilnahme der Person am Zielsetzungs- und Behandlungsprozess.

# Der Begriff „Hauptmuskelgruppe“ wurde ersetzt durch „größere Muskelgruppe“. Größere Muskelgruppen umfassen Bein‑, Arm‑, Brust‑, Bauch‑, Rücken- und Schultermuskulatur.

# Die Bewegungsempfehlungen für Gesunde der WHO/ACSM wurden 2011 publiziert und 2020 erneut überarbeitet [5, 22]. Da die Empfehlungen von 2011 die Basis für die EULAR Bewegungsempfehlungen darstellte, dienten sie auch in dieser Übersetzung weiterhin als Grundlage. Die erfolgten Aktualisierungen sind in der Tab. 2 deshalb noch nicht berücksichtigt, aber als Anmerkungen (*) in die Legende eingefügt. So ist in den aktuellen WHO-Empfehlungen formuliert, dass kardiovaskuläres Training nicht mehr in Einheiten von mindestens 10 min erfolgen muss, sondern jede Aktivität unabhängig ihrer Dauer zählt. Zudem wurden die empfohlenen Zeiteinheiten (Dauer) für kardiovaskuläres Training für moderate Intensität von 150 auf 150–300 min und für hohe Aktivität von 75 auf 75–150 min präzisiert.

Insgesamt wurde die Übersetzung zu Tab. 2 von den deutschsprachigen Teilnehmenden als stimmig befunden.

## Diskussion

Die übersetzten EULAR Bewegungsempfehlungen wurden von der Experten*innengruppe mit Teilnehmenden aus Deutschland, Österreich und der Schweiz sprachlich validiert und führten zu einer finalen Version der Übersetzung, die von allen beteiligten Experten*innen aus den 3 Ländern mit einem hohen Grad der Zustimmung bewertet wurde. Es wurde bei der Bearbeitung der Übersetzung versucht, zwischen den Experten*innen der 3 Länder einen größtmöglichen Konsens zu erreichen. In einigen Punkten wurden Kompromisse diskutiert und gefunden, da in den einzelnen Ländern zum Teil unterschiedliche Begrifflichkeiten bestehen, wie z. B. die Begriffe Gesundheitsdienstleister*innen bzw. Gesundheitsfachberuf. Die Möglichkeiten der Implementierung der Empfehlungen in den Alltag, Reglementierungen zur Verordnung und die Art und Weise der Interaktionen der einzelnen Fachdisziplinen untereinander weisen länderspezifische Unterschiede auf und wurden deshalb nicht in die übersetzte Version aufgenommen, obwohl solche Präzisierungen gewünscht worden waren.

In den 10 Empfehlungen werden neben der Integration der Förderung von körperlicher Aktivität in die Standardversorgung auch wichtige spezifische Hinweise für Patient*innen und Gesundheitsdienstleister*innen formuliert, beispielsweise wie und in welchem Umfang körperliche Aktivität durchgeführt werden soll, inklusive der Nennung von Barrieren und Förderfaktoren.

Die Grundlage der EULAR Bewegungsempfehlungen von 2018 basiert auf den international anerkannten WHO/ACSM Bewegungsempfehlungen für Gesunde von 2011 [8]. Da die WHO-Empfehlungen 2020 überarbeitet worden sind, wurden einige spezifische Punkte, wie z. B. die Änderungen der empfohlenen Zeiteinheiten, in der hier vorliegenden deutschen Übersetzung als Anmerkungen mit aufgenommen. Allgemein gelten die WHO-Empfehlungen von 2020 als derzeitige Referenz.

Die EULAR Bewegungsempfehlungen liefern genaue Hinweise für den Umgang, die Intensität, Dauer sowie Art und Weise der körperlichen Aktivität und gibt zudem Orientierungshilfen zur Umsetzung und Gestaltung von körperlicher Aktivität bei Patient*innen mit RA/SpA/HKA. Dass sie nun auf Deutsch vorliegen, stellt unserer Ansicht nach eine Chance für die Anwendung und Implementierung der EULAR Bewegungsempfehlungen im deutschsprachigen Raum dar. Daher sind wir zuversichtlich, dass diese Empfehlungen für Patient*innen und Gesundheitsdienstleister*innen einschließlich Rheumatolog*innen und anderen Spezialist*innen sowie PTs, ETs, Pflegefachpersonen und MFAs nutzbar sein werden. Die Übersetzung alleine wird keine automatische, flächendeckende Implementierung zur Folge haben. Nationale Implementierungsstrategien für D/AT/CH sollten entwickelt werden.

## Fazit für die Praxis

- Körperliche Aktivität ist ein wichtiger Baustein in der Behandlung von Patient*innen mit entzündlich-rheumatischen Erkrankungen und degenerativen Erkrankungen, da sie einen positiven Einfluss auf den Krankheitsverlauf hat.
- Die Bewegungsempfehlungen für Gesunde gelten genauso für Menschen mit RA/SpA/HKA, weil sie sicher angewendet werden können.
- Die EULAR Bewegungsempfehlungen für Menschen mit RA/SpA/HKA sollen die Förderung von körperlicher Aktivität für die behandelnden Gesundheitsdienstleister*innen sowie die Durchführung von körperlicher Aktivität von RA/SpA/HKA betroffenen Menschen stärken. Sie sollen eine Orientierungshilfe für Verschreibung, Anleiten und Durchführen von körperlicher Aktivität inklusive dem Umgang mit Barrieren und Förderfaktoren geben.
- Die hier vorgestellte Übersetzung der EULAR Bewegungsempfehlungen soll die Nutzung und Etablierung dieser Empfehlungen im deutschsprachigen Raum fördern.
- Eine Lai*innenversion in patient*innenverständlicher Sprache wird ebenfalls vorgestellt.
